# CCR5+ T-Cells Homed to the Liver Exhibit Inflammatory and Profibrogenic Signatures in Chronic HIV/HCV-Coinfected Patients

**DOI:** 10.3390/v13102074

**Published:** 2021-10-14

**Authors:** Shikha Shrivastava, Shyam Kottilil, Kenneth E. Sherman, Henry Masur, Lydia Tang

**Affiliations:** 1 Laboratory of Adjuvant and Antigen Research, US Military HIV Research Program, Walter Reed Army Institute of Research, Silver Spring, MD 20910, USA; sshrivastava@hivresearch.org; 2 Institute of Human Virology, University of Maryland School of Medicine, Baltimore, MD 21201, USA; SKottilil@ihv.umaryland.edu; 3 Program in Oncology, Marlene and Stewart Greenebaum Comprehensive Cancer Center, University of Maryland, Baltimore, MD 21201, USA; 4 Division of Digestive Diseases, University of Cincinnati, Cincinatti, OH 45267, USA; shermake@ucmail.uc.edu; 5 National Institutes of Health, Bethesda, MD 20892, USA; hmasur@nih.gov

**Keywords:** HIV, hepatitis C, fibrosis, hepatic fibrogenesis, CCR5, HIV/HCV coinfection

## Abstract

Liver fibrosis is accelerated in patients coinfected with hepatitis C virus and human immunodeficiency virus (HIV), compared with HCV monoinfected patients, although the underlying mechanisms are unknown. We hypothesize that T cells expressing the HIV co-receptor, chemokine receptor 5 (CCR5), preferentially migrate to the inflamed liver and contribute to enhanced fibrogenesis. We compared the peripheral and intrahepatic CCR5 expression on CD4+ and CD8+ T cells in 21 HIV/HCV-coinfected patients with 14 chronic HCV monoinfected patients. Using 12-color flow cytometry, phenotypic and functional characterization of CCR5+ and negative cells pre- and post-stimulation with HCV genotype specific overlapping pooled peptides was conducted. Patients with HIV/HCV coinfection had significantly more CD4+CCR5+ and CD8+CCR5+ T cells in the liver as compared with peripheral blood (*p* = 0.0001 for both). Compared with patients with HCV monoinfection, patients with HIV/HCV coinfection also had fewer peripheral CD4+CCR5+ and CD8+CCR5+ T cells (*p* = 0.02, *p* = 0.001 respectively), but more intrahepatic CD4+CCR5+ and CD8+CCR5+ cells (*p* = 0.0001 for both). Phenotypic analysis of CCR5+ sorted cells demonstrated an increased expression of markers of exhaustion, senescence, immune activation and liver homing (PD1, CD57, CD38, HLADR, and CXCR3). Post-stimulation with HCV peptides, CCR5+ T cells secreted more proinflammatory and profibrogenic cytokines and chemokines rather than antiviral cytokines. Phenotypic and functional analyses of CCR5+ T cells in HIV/HCV-coinfected patients revealed a pathogenic role for CCR5+ T cells in hepatic fibrogenesis. These cells are functionally proinflammatory, pro-fibrogenic and preferentially accumulate in liver, accelerating fibrosis. These findings suggest that targeting CCR5 may be a therapeutic strategy for be ameliorating liver fibrosis.

## 1. Introduction

Human immunodeficiency virus (HIV) and hepatitis C virus (HCV) infections are major global public health problems. Due to shared modes of transmission, the prevalence of HCV co-infection in people living with HIV in the United States is high, ranging between 17 and 37% [1,2]. HIV/HCV coinfection increases the risk of developing end-stage liver disease or cirrhosis by 2.9 times and accelerates the development of liver fibrosis as compared with HCV monoinfection [3]. In countries where antiretroviral therapy (ART) is available and utilized for HIV, rates of hepatic decompensation among HIV/HCV-coinfected patients are higher, compared with patients with HCV monoinfection [4,5,6,7].

The mechanism for accelerated liver disease in coinfection has yet to be fully defined, but immune dysregulation has been implicated [8,9,10]. The CC chemokine receptor 5 (CCR5) belongs to a large family of chemokine receptors that orchestrate the recruitment, migration and activation of mononuclear cells in liver inflammation. Present on CD4 T cells, CCR5 is a co-receptor for GP-120, a protein present on the envelope of the HIV virus and essential for HIV cell entry [11,12]. CCR5 is also expressed on activated hepatic stellate cells (HSC, non-parenchymal cells that undergo phenotypic transition to myofibroblast-like cells during hepatic injury and are the main source of intrahepatic fibrotic tissue) [13]. CCR5 and its ligands (MIP 1-alpha [CCL3], MIP 1-beta [CCL4], and RANTES [CCL5]) are potential key players in hepatic inflammation and fibrosis [14,15]. Recently, reduction in non-invasive (serum-based) measures of liver fibrosis with dual CCR2 and 5 blockade was reported among patients with HIV [16] and among patients with non-alcoholic steatohepatitis without HIV [17].

The intrahepatic enrichment of CCR5+ lymphocytes in HCV infection has been reported, suggesting a role for inflammatory cell migration to the liver mediated by CC chemokines [18]. In this study, we explored the impact of HIV/HCV coinfection on CCR5-mediated hepatic fibrogenesis. We hypothesized that HIV/HCV coinfection augments the preferential migration of CCR5+ T cells to the inflamed liver, enhancing fibrogenesis. We compared blood samples from patients with HIV/HCV coinfection, and HIV and HCV monoinfection. We first characterized the differences in peripheral and intrahepatic CCR5+ T cell populations. Then we compared the differences in phenotypic, homing, functional and secretory characteristics associated with CCR5 positivity in patients with HIV/HCV coinfection and compared these with HCV and HIV monoinfection.

## 2. Materials and Methods

### 2.1. Participants

Research samples used for this study were obtained from participants enrolled in prospective natural history studies at the National Institute of Allergy and Infectious Diseases (NIAID) and the Institute of Human Virology at the University of Maryland School of Medicine. Samples included were selected based on the following criteria: adults with HIV mono-, HCV mono- or HIV/HCV coinfection. Participants living with HIV were taking ART with suppressed HIV viral load (<50 copies/mL). Participants with HCV were naïve to HCV treatment. Samples from participants with hepatitis B or delta infection were excluded. HCV and HIV infections were confirmed by HCV RNA and HIV assays (Roche Amplicor, Roche Molecular Diagnostics, Pleasanton, CA, USA). Fibrosis staging was done by FibroScan^®^ (Echosens, MA, USA), with cirrhosis (F4) defined as greater than 12.5 kilopascals, liver biopsy or FibroSure^®^.

Peripheral blood mononuclear cells (PBMC) were analyzed from 10 HIV monoinfected, 10 HCV monoinfected, and 10 HIV/HCV-coinfected patients (PBMC only cohort). A separate group of 35 patients (21 HIV/HCV-coinfected, and 14 HCV monoinfected) had previously undergone paired liver biopsies and PBMC sampling (liver biopsy cohort) and their data analyzed in this study.

The NIAID and University of Maryland Medical Center Institutional Review Boards approved the parent natural history studies (04-I-0086, HP-00063191) and written informed consent was obtained from all participants.

### 2.2. Immunophenotyping and CCR5 Cell Surface Density Determination in Peripheral Blood Mononuclear Cells (PBMCs)

PBMCs were isolated by density gradient centrifugation. Cells were counted by trypan blue exclusion and stored in liquid nitrogen until use. Frozen PBMCs were thawed and stained with the fluorochrome-conjugated monoclonal antibodies (Supplementary Table S1) for 30 min at 4 °C using standard protocol as described previously [19]. The mean number of CCR5 molecules at the surface of each CD4+ and CD8+ T cell was determined and extrapolated to determine surface CCR5 density as described by Reynes et al. (detailed in Supplementary File S1) [20].

### 2.3. Isolation of Liver Infiltrating Lymphocytes (LILs) and Intrahepatic CCR5 Frequency Determination

LILs were isolated from the liver biopsy samples by mechanical dissociation (detailed in Supplementary File S1) [21]. LILs and fresh paired PBMCs were immediately stained with fluorochrome-conjugated antibodies (Supplementary Table S1) and frequency of liver and peripheral CCR5 expressing CD4+ and CD8+ T cells determined.

### 2.4. HCV Peptide Reconstitution

Genotype 1a or 1b HCV 15- to 18-mer peptides with 11 or 12 amino acid overlaps spanning the entire HCV polyprotein (BEI Resources, NIAID, NIH: Peptide Array, Hepatitis C Virus) were reconstituted in 5% sterile dimethylsulphoxide (DMSO) and pooled consecutively into twenty-one groups. All twenty-one groups were pooled together to make a peptide pool covering the entire HCV genome. Peptides were aliquoted and stored at −80 °C until use.

### 2.5. Cell Sorting and HCV Peptide Specific T Cell Functions

CD4+ and CD8+ T cells were isolated by negative selection and sorted for CCR5+ and CCR5 negative cells using a BD FACS ARIA cell sorter (gating strategy is provided in Supplementary Figure S2). Antigen specific cytokine secretion was assessed by multi-parameter intracellular cytokine staining. Sorted cells were incubated for 5 days at 37 °C in 5% CO_2_ with either genotype specific overlapping HCV peptide pool (2 µg/mL/peptide) covering the entire genome, or phorbol-12-myristate-13-acetate (PMA) (2.5 μg/mL) and Ionomycin (0.5 μg/mL) (Sigma) (positive control) or medium alone (negative controls). At day 4, cells were restimulated and incubated for another 2 h at 37 °C in 5% CO_2_. Brefeldin A (1 μg/mL, Sigma) and 1 μL of monensin (1 μg/mL, Golgi-Stop, BD Biosciences) was then added and the cells incubated for an additional 10 h at 37 °C in 5% CO_2_. Cells were then harvested and stained using the panel of surface and intracellular antibodies (Supplementary Table S1) following standard procedure as previously described [19]. Detailed methods are provided in Supplementary File S1.

### 2.6. Analysis of Cytokine Production by Multiplex ELISA

To estimate cytokine and chemokine secretion by CCR5+ and CCR5 negative T cells, frozen cellular supernatants (after 96 h of HCV peptide stimulation) were thawed and analyzed in a 34-plex assay according to manufacturer recommendations (Human ProcartaPlex™ Panel; Cat.No. EPX340-12167-901; ThermoFisher scientific, Carlsbad, CA, USA) with Luminex instruments (Bio-Plex^®^ 200 systems; Bio-Rad, Hercules, CA, USA).

### 2.7. Statistical Analysis

Differences in the frequency, median fluorescence intensity (MFI), density of CCR5 on peripheral T cells between the two groups, and differences in the frequency of CCR5 expression on T cells between paired peripheral and liver samples were evaluated by non-parametric Mann–Whitney test. Correlation between hepatic CCR5 frequencies and fibrosis stage was determined by Pearson correlation analysis. All statistical analysis was conducted using Graph Pad Prism version 6.0.

## 3. Results

### 3.1. Patient Characteristics

Baseline characteristics are summarized in Table S1. In the PBMC-only cohort, patients from each group were similar in age and liver disease stage. In the liver biopsy group, the HIV/HCV group had more females. CD4 count was higher in the HCV monoinfected group.

### 3.2. Lower Frequency of CCR5+ CD4 and CD8 T Cells in the Peripheral Blood of Patients with HIV/HCV Coinfection Compared to HCV Monoinfection

**Figure 1:**
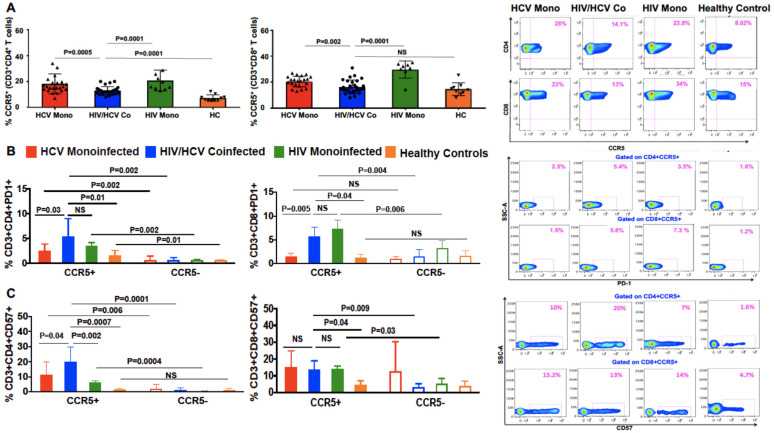
Peripheral blood analysis of CCR5+ and CCR5 negative CD4+ and CD8+ T cells in patients with HIV/HCV coinfection and HCV monoinfection. Flow cytometry plots from a patient and bar graphs with HCV monoinfection, HIV/HCV coinfection, HIV monoinfection and healthy controls demonstrating the differences in the percentages of (**A**) CCR5 on CD4 and CD8 T cells (**B**) Percentages of PD1 on CD4+CCR5+ and CD8+CCR5+ T cells (**C**) Percentages of CD57 on CD4+CCR5+ and CD8+CCR5+ T cells. Data are expressed as (Mean ± SEM) and differences in percentage of T cells expressing different markers between the two groups were analyzed using non-parametric Man-Whitney test in Graph Pad Prism version 6. *p* value less than 0.05 was considered as significant.

All patient groups had higher frequencies of CD4+CCR5+ T cells, compared with healthy controls. However, HIV/HCV coinfection had significantly less CCR5+ cells compared with both HCV and HIV monoinfection (Figure 1A).

No differences in CCR5 MFI and density (molecules/cell) were observed on CD4+ and CD8+ T cells (Supplementary Figure S1). Therefore, in our study HIV/HCV coinfection was associated with an overall peripheral reduction—and not due to a reduction in surface expression per cell—of CCR5 expressing CD4+ and CD8+ T cells compared with HCV and HIV monoinfection.

### 3.3. Peripheral CCR5+ T Cells from HIV/HCV-Coinfected Patients Express More PD1 and CD57 Than Cells from HCV Monoinfected Patients

PD1 and CD57 are important markers for T cell exhaustion and senescence. CD57+ cells are proliferation incompetent and terminally differentiated cells that are susceptible to apoptosis. Higher expression of CC57+ cells are seen in HIV and other chronic viral infections [22,23,24,25]. We analyzed the phenotypic differences between the CCR5+ and CCR5 negative CD4+ and CD8+ T cells with respect to PD1 and CD57 expression. In both HIV mono- and HIV/HCV-coinfected patients, more CCR5+ cells expressed PD1 as compared with CCR5 negative cells (Figure 1B, *p* = 0.002 for both). In HCV monoinfection, this difference was statistically significant on the CD4+ cells only (CD4: *p* = 0.002; Figure 1B). The overall expression of PD1, however, was significantly higher in the coinfected group, compared with the HCV monoinfected group (CD4: *p* = 0.03; CD8: *p* = 0.005; Figure 1B) and healthy control group (CD4: *p* = 0.01; CD8: *p* = 0.04; Figure 1B) while no differences were observed when comparing HIV/HCV coinfection with HIV monoinfection. Among patients with HIV/HCV coinfection, CD57 expression was also higher in CCR5 expressing CD4+ and CD8+ T cells compared with CCR5 negative cells (CD4: *p* = 0.0001; CD8: *p* = 0.009; Figure 1C) and similarly among patients with HIV monoinfection (CD4: *p* = 0.0004; CD8: *p* = 0.03; Figure 1C). Among patients with HCV monoinfection, however, increased CD57 expression was seen only in CD4+CCR5+ T cells, compared with CCR5 negative CD4+ cells (*p* = 0.006; Figure 1C), and not in CD8+ cells. Furthermore, in the CD4+ cell population, the frequency of CD4+CCR5+CD57+ T cells was higher in HIV/HCV-coinfected patients as compared with HCV and HIV monoinfected patients and healthy controls (Figure 1C). Therefore, in peripheral blood, HIV/HCV coinfection was associated with increased CCR5+ T cells with an exhausted and terminally differentiated phenotype compared to monoinfected patients.

### 3.4. Increased Co-Expression of Markers of Chronic Immune Activation, HLA-DR and CD38, on CCR5+ Compared to CCR5 Negative T Cells in Coinfected Patients

**Figure 2:**
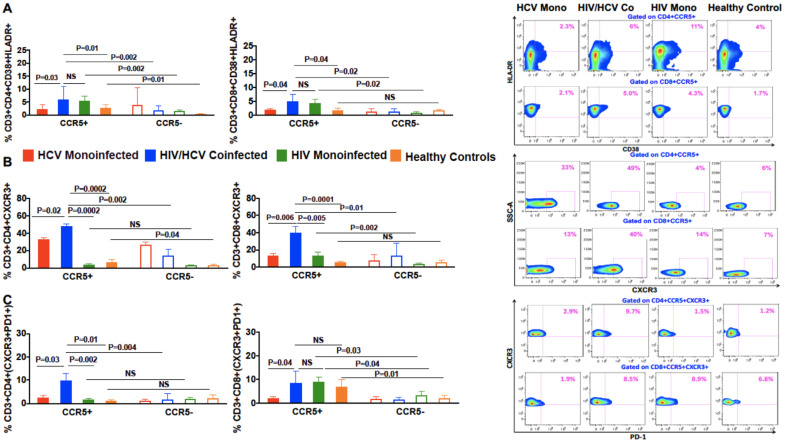
Differences in the frequencies of CD38/HLADR, CXCR3 and PD1+CXCR3+ on CCR5+ and CCR5 negative CD4+ and CD8+ T cells in patients with HIV/HCV coinfection and HCV monoinfection. Flow cytometry plots of a patient and bar graphs with HCV monoinfection, HIV/HCV coinfection, HIV monoinfection and healthy controls demonstrating the differences in the percentages of (**A**) CD38+HLADR+ on CD4+CCR5+ and CD8+CCR5+ T cells (**B**) CXCR3 on CD4+CCR5+ and CD8+CCR5+ T cells (**C**) CXCR3+PD1+ on CD4+CCR5+ and CD8+CCR5+ T cells. Data are expressed as (mean ± SEM) and differences in the percentage of T cells expressing different markers between the two groups were analyzed using a non-parametric Mann–Whitney test in Graph Pad Prism version 6. The Wilcoxon matched-pair signed rank test was used for analyzing the differences between the CCR5+ and CCR5 negative T cells within the same group. *p* values less than 0.05 were considered significant.

We detected no difference in the expression of HLA-DR and CD38 between CCR5+ and CCR5 negative T cells in HCV monoinfected patients. However, expression of CD38+ HLA-DR+ was increased on CCR5+ T cells compared with CCR5 negative T cells in the HIV/HCV-coinfected (CD4: *p* = 0.002; CD8: *p* = 0.02; Figure 2A) and HIV monoinfected groups (CD4: *p* = 0.002; CD8: *p* = 0.02; Figure 2A). CCR5+ T cells of coinfected patients also had a higher frequency of HLA-DR and CD38 dual expression as compared with HCV monoinfected patients (CD4: *p* = 0.03; CD8: *p* = 0.04; Figure 2A). Thus, CCR5+ T cells in HIV/HCV-coinfected patients expressed an activated phenotype, compared to HCV monoinfected patients.

### 3.5. In Patients with HIV/HCV Coinfection, Peripheral CCR5+ T Cells Had a Higher Frequency of the T Cell Trafficking Receptor, CXCR3, Compared to CCR5 Negative T Cells

Next, we analyzed the homing properties of peripheral CCR5+ T cells from the different patient groups by evaluating the expression of CXCR3, which plays an important role in T cell trafficking and functions. We found that CCR5 expressing CD4+ and CD8+ T cells from coinfected patients had a higher percentage of CXCR3 expression than CCR5 negative cells (CD4: *p* = 0.002; CD8: *p* = 0.01; Figure 2B). The frequency of CCR5+ cells from coinfected patients that expressed CXCR3 was also higher than CCR5+ cells from HCV monoinfected patients (CD4: *p* = 0.02; CD8: *p* = 0.006; Figure 2B), HIV monoinfected patients (CD4: *p* = 0.0002; CD8: *p* = 0.005; Figure 2B) and healthy controls (CD4: *p* = 0.0002; CD8: *p* = 0.0001; Figure 2B). CD4+CCR5+ and CD8+CCR5+ T cells from coinfected patients also had greater CXCR3+PD1+ co-expression, compared with CCR5 negative (CD4: *p* = 0.004, CD8: 0.03, Figure 2C). CXCR3+PD1+ co-expression was also higher in CD4+CCR5+ T cells from patients with HIV/HCV as compared with CD4+CCR5+ T cells from HIV and HCV monoinfected patients (CD4: *p* = 0.002 and 0.03; Figure 2C) and healthy controls (CD4: *p* = 0.01; Figure 2C). These results suggest that CCR5+ T cell with an exhausted phenotype migrate to the liver, contributing to lower peripheral distribution.

### 3.6. In HIV/HCV Coinfection, HCV-Specific CCR5+ T Cells Secrete Less IL-2 and IFN-γ, and More TGF-β as Compared with HCV Monoinfection

**Figure 3:**
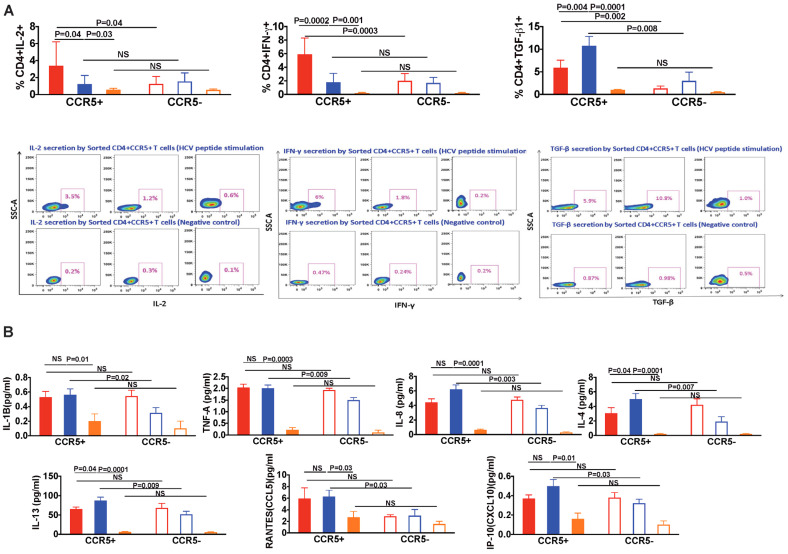
Functional analysis of HCV specific CCR5+ and CCR5 negative CD4+ T cells in HIV/HCV coinfection and HCV monoinfection. (**A**) Flow cytometry figures of a patient and bar graphs with HCV monoinfection, HIV/HCV coinfection and healthy controls demonstrating the differences in the functional response of CD4+CCR5+ after in-vitro stimulation with HCV peptides and media (served as negative control) in terms of IL-2, IFN-gamma and TGF-β production. (**B**) Bar graphs demonstrating the differences in the secretion of pro-Inflammatory (IL-1β, TNF-A, IL-8) and pro-fibrotic (IL-4 and IL-13) cytokines and Chemokines (RANTES and IP-10) by CCR5+ T cells in HIV/HCV coinfection, HCV monoinfection and healthy controls. Data are expressed as (mean ± SEM) and the Wilcoxon matched-pair signed rank test was used to evaluate the functional differences between CCR5+ and CCR5 negative T cells within the same group and to analyze the differences between the two different groups non-parametric Mann–Whitney test was used. *p* values less than 0.05 were considered significant.

As previously demonstrated, CCR5+ T cells from HIV/HCV-coinfected patients appear to be activated, terminally differentiated, exhausted and express CXCR3 indicating migration to the inflamed liver. However, this does not attribute a pathogenic role for these cells. Hence, we evaluated and compared the secretory functional response of CCR5+ and CCR5 negative T cells. After in-vitro stimulation with a panel of pooled overlapping HCV peptides spanning the entire HCV genome, intracellular cytokine staining for cytokines association with antiviral function and fibrogenesis, IL-2, IFN-γ, and TGF-β, was performed. CD4+CCR5+ T cells from HIV/HCV-coinfected patients produced less IL-2 and IFN–γ compared with HCV monoinfected patients (*p* = 0.04 and 0.0002, respectively, Figure 3A). Rather, these cells from HIV/HCV-coinfected patients favored production of TGF-β compared with CD4+CCR5 negative T cells (*p* = 0.008, Figure 3A). TGF-β production by CD4+CCR5+ T cells was also higher in HIV/HCV coinfection, compared with HCV monoinfection and healthy controls (*p* = 0.004 and 0.001, respectively, Figure 3A). These findings indicate that HCV-specific CCR5+ T cells have decreased antiviral function and may favor a profibrogenic microenvironment.

### 3.7. Pro-Inflammatory and Pro-Fibrotic Cytokines and Chemokines Are Highly Secreted by CCR5+ T Cells in HIV/HCV Coinfection

Following up our previous findings, cellular supernatants were analyzed for various pro-inflammatory and pro-fibrotic cytokines and chemokines in a multiplex assay. In HIV/HCV coinfection, CD4+CCR5+ T cells secreted more pro-inflammatory cytokines IL-1β, TNF-α, IL-8 than CCR5 negative cells (IL-1β: *p* = 0.02, TNF-α: *p* = 0.009, IL-8: *p* = 0.003; Figure 3B). Similarly, CD4+CCR5+ T cells from coinfected patients secreted more pro-fibrotic cytokines IL-4 and IL-13 [26] than CCR5 negative cells (IL-4: *p* = 0.007, IL-13: *p* = 0.009; Figure 3B). Increased IL-4 and IL-13 secretion from CD4+CCR5+ T cells was also seen in coinfection as compared with monoinfection (*p* = 0.04 for both) and healthy controls (See Figure 3B). We also evaluated IP-10/CXCL10 and RANTES/CCL5 (pro-fibrotic chemokines) secretion. In HIV/HCV coinfection, both were increased from CD4+CCR5+ T cells, compared with CCR5 negative cells (*p* = 0.03 for both IP-10(CXCL10) and RANTES Figure 3B), and compared with healthy control (IP-10(CXCL10) *p* = 0.01, RANTES *p* = 0.03).

### 3.8. Increased Frequency of Intrahepatic CCR5+ T Cells Compared to Periphery, Correlated with Increased Fibrosis

**Figure 4:**
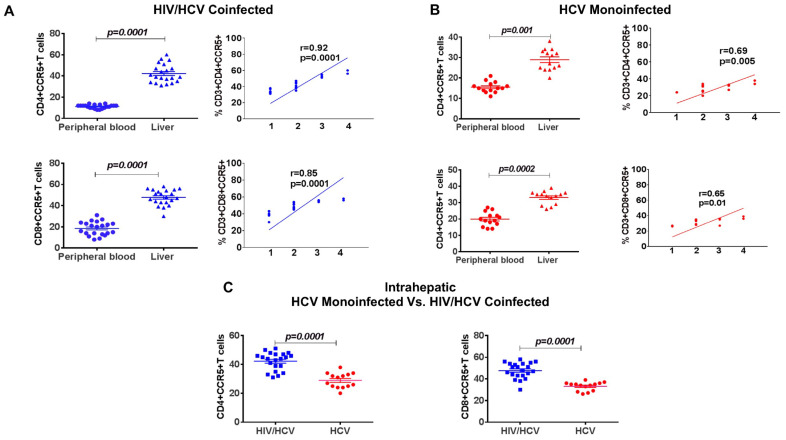
Peripheral blood vs. liver analysis in HIV/HCV coinfection and HCV monoinfection. Dot plot graphs showing the differences in the frequencies of CCR5 on CD4+ and CD8+ T cells in the peripheral and intrahepatic compartment and XY scatter plots showing the correlation (Pearson correlation analysis) between CCR5 frequencies in the liver and fibrosis stage in (**A**) HIV/HCV-coinfected patients (n = 21) and (**B**) HCV monoinfected (n = 14) patients who underwent paired liver and peripheral blood sampling. (**C**) The intrahepatic differences in the frequencies of CCR5 on CD4+ and CD8+ T cells in the 14 monoinfected and 21 HIV/HCV-coinfected patients.

Next, we compared the peripheral and intrahepatic CCR5+ LIL frequencies in a separate cohort of 14 HCV monoinfected and 21 HIV/HCV-coinfected patients who underwent paired liver and peripheral blood sampling. Overall, CD4+CCR5+ and CD8+CCR5+ T cells were enriched in the liver, compared to periphery in both groups (CD4: *p* < 0.0001; CD8: *p* < 0.0001; Figure 4A). Intrahepatic CCR5+ T cell populations were larger in coinfection compared to monoinfection (CD4: *p* < 0.0001; CD8: *p* < 0.0001; Figure 4C). Finally, we observed a positive correlation between intrahepatic CCR5+ frequency and liver fibrosis in both monoinfected (CD4: r = 0.69 *p* = 0.005; CD8: r = 0.65 *p* = 0.01; Figure 4B) and coinfected patients (CD4: r = 0.92 *p* = 0.0001; CD8: r = 0.85 *p* = 0.0001; Figure 4A).

## 4. Discussion

**Figure 5:**
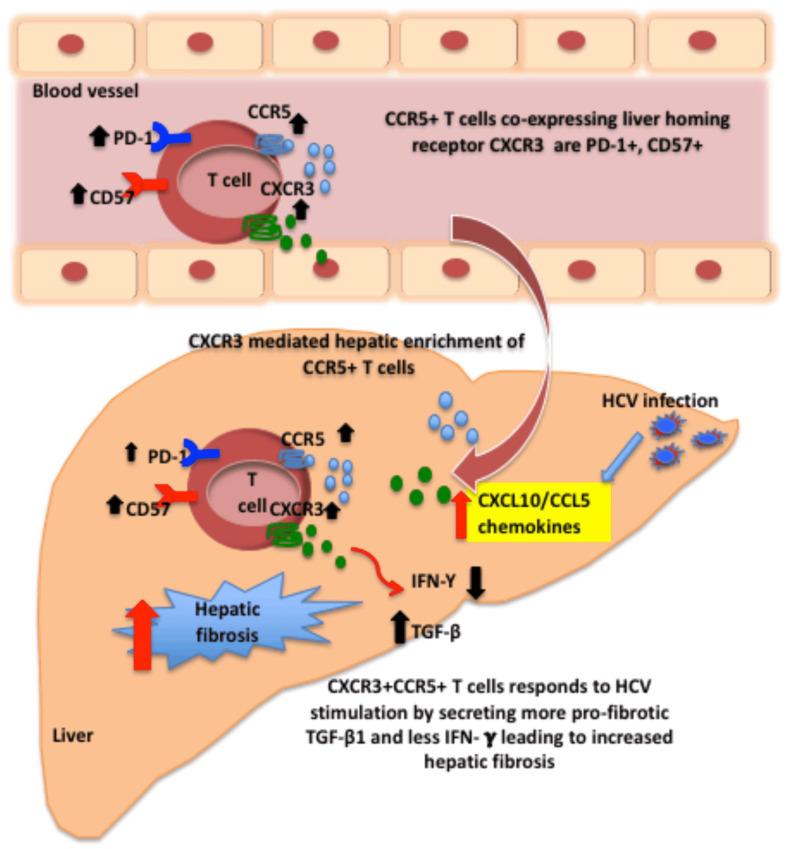
Graphical summary of this study. Hepatitis C virus infection in the liver of HIV infected patients stimulates the production of chemokines including CCL5 (RANTES), IP-10 (CXL10), which induces migration of CCR5+CXCR3+ T cells from the peripheral blood to the liver. These CCR5+CXCR3+ T cells infiltrating into the liver are chronically activated, terminally differentiated and have an exhausted phenotype. Upon HCV peptide stimulation, these cells secrete more pro-fibrogenic cytokine (TGF-β) rather than antiviral cytokines (IFN-γ and IL-2), which may favor a profibrogenic intrahepatic microenvironment in HIV/HCV coinfection, causing advanced liver disease.

Our study presents a possible CCR5-based mechanism for the enhanced hepatic fibrogenesis observed in HIV/HCV-coinfected patients. Our findings suggest greater hepatic migration and corresponding intrahepatic enrichment of pro-fibrogenic TGF-β secreting CCR5+ T cells in HIV/HCV coinfection, compared to HCV monoinfection. These findings provide valuable insights into the pathogenesis of CCR5 as a potential therapeutic target for ameliorating liver fibrosis in HIV/HCV-coinfected patients (Figure 5).

CCR5 and its chemokine ligand (CCL5) has a critical role in hepatic fibrogenesis [14]. We hypothesize that this occurs through cross talk between CCR5+ T cells with profibrogenic and proinflammatory function, and hepatic stellate cells (HSC), which are the central mediator in fibrotic response within the liver. Activated HSCs secrete cytokines and chemokines that contribute to the proinflammatory and profibrotic milieu. CCR5 has been observed to play a role in HSC-mediated pro-fibrogenesis through indirect induction of CC-chemokines, and CCR5 antagonism has been shown to result in decreased RANTES-induced HSC migration [13,14]. Other studies have shown intrahepatic enrichment of CCR5 in HCV infected livers as compared with un-infected, and in cirrhotic livers, compared with non-cirrhotic [18,27,28], and in a murine model of acute liver failure, massive hepatic infiltration by non-specific T cells expressing CCR5 and the upregulation of CCR5 mRNA, chemokines MIP 1-alpha and beta, and RANTES was reported [29]. In animal studies, acute liver failure and the regression of liver fibrosis was attenuated by antibody-mediated antagonism of CCR5 and RANTES. Additionally, mice, deficient in CCR5, demonstrated significantly less hepatic fibrosis [29,30]. CXCR3 and its associated chemokines CXCL9, CXCL10 (also known as IP-10 and CXCL11) play a key role in the hepatic recruitment of lymphocytes during HCV infection, with expression thereof being positively correlated with liver inflammation [31,32]. Our study further support these prior studies by showing that in HIV/HCV coinfection, CCR5+ T cells are highly sequestrated in liver and exhibit fibrogenic, rather than antiviral, responses, and may contribute to accelerated fibrosis in HIV/HCV coinfection.

There are several reports supporting a CCR5-mediated mechanism for hepatic inflammation and fibrosis in HCV infection; however, there has been little reported on how HIV coinfection affects these pathways. Our findings indicate that the more aggressive natural history of liver disease among patients with HIV/HCV coinfection may, in part, be due to CCR5-mediated T cell dysregulation and dysfunction.

CCR5 antagonism may represent a therapeutic strategy for ameliorating hepatic fibrosis in HIV/HCV coinfection, as suggested by studies demonstrating lower rates of progression of liver fibrosis on patients receiving maraviroc, a small molecule inhibitor of CCR5 is used as part of ART [33,34]. A dual CCR5/CCR2 antagonist, cenicriviroc, has recently been shown to limit fibrosis in animal models and humans [35,36]. Leronlimab is an investigational humanized IgG4 monoclonal antibody that blocks CCR5 and is currently in phase two clinical trial for the treatment of non-alcoholic steatohepatitis (NCT04521114).

The strengths of this study include the analysis of paired liver biopsy and peripheral blood samples from well-characterized cohorts. Our study was limited by the small number of participants and cross-sectional design. A longitudinal study with a larger number of participants at different stages of liver fibrosis would provide more comprehensive insight into the phenotypic and functional changes within the CCR5+ and CCR5 negative T cell subsets and how these change with progression in liver disease. Our study would have been further strengthened by the evaluation of cytokines in plasma, and cytokine transcripts in the liver. However, due to sampling limitations, these were not possible. In conclusion, phenotypic and functional analysis of CCR5+ T cells support their role in the pathogenesis of accelerated hepatic fibrogenesis in HIV/HCV coinfection. Targeting CCR5 may be a therapeutic strategy for liver fibrosis in patients with HIV/HCV coinfection.

## Data Availability

All the data supporting the findings of this study can be found within the paper in Table S1, Figure 1, Figure 2, Figure 3, Figure 4 and Figure 5, Supplementary Figures S1 and S2.

## Supplementary Materials

The following are available online at https://www.mdpi.com/article/10.3390/v13102074/s1, Supplementary File S1: Detailed Methods, Supplementary Table S1: List of antibodies used for immunophenotyping of PBMC s and LILs, Supplementary Figures S1 and S2.
