# Supplementary material for: CCR5+ T-Cells Homed to the Liver Exhibit Inflammatory and Profibrogenic Signatures in Chronic HIV/HCV-Coinfected Patients
Source: Viruses. 2021 Oct 14;13(10):2074. doi: 10.3390/v13102074 (PMC8539814; doi:10.3390/v13102074)
Supplement: Supplementary file 1 [file viruses-13-02074-s001.zip › viruses-1373701-supplementary final.pdf]

viruses

MDPI

Supplementary file S1: Detailed Methods

Immunophenotyping and CCR5 cell surface density determination in peripheral blood mononuclear cells (PBMCs)

PBMCs were isolated using Ficoll-Paque (GE Health Care Life Sciences, Life Technologies, Grand Island, NY, USA) and density gradient centrifugation method. Cells were counted by trypan blue exclusion and stored in liquid nitrogen until use. Frozen PBMCs were thawed and stained with the fluorochrome-conjugated monoclonal antibodies listed in supplementary Table 1 for 30 min at 4 °C using standard protocol as described previously [13]. For CCR5 surface density determination the mean number of CCR5 molecules at the surface of each CD4+ and CD8+ T cell was determined and extrapolated to density of CCR5 as described by Reynes et al. [14]. Briefly, fluorescence was measured using the Quantiquest system (BD Biosciences), and a regression line produced from a series of Quantibrite-PE bead standards (BD Biosciences). The mean number of CCR5 molecules expressed on the surface of the cells labeled was determined from the FL-2 value (PE-labeled antibody) using a linear regression and taking into account the 1:1 PE antibody ratio for each antibody used in our assay.

Isolation of liver infiltrating lymphocytes (LILs) and Intrahepatic CCR5 frequency determination

Paired liver biopsies and PBMCs were collected from 14 HCV monoinfected and 21 HIV/HCV coinfected participants. PBMCs were isolated as described above. LILs were isolated from the liver biopsy samples by mechanical dissociation [15]. First, liver tissue was obtained through ultrasound-guided percutaneous 18-guage-core biopsy, and immediately placed in 5ml lymphocyte medium (RPMI supplemented with 10% fetal bovine serum [FBS], 1% penicillin and streptomycin, 1mM HEPES and 20 micromolar of 2-mercaptoethanol, all from Life Technologies) and processed within 30 min of collection. The biopsy was first rinsed in Hank's solution (2% FBS and 1% EDTA) to remove exogenous blood, and then minced and homogenized according to manufacturer's instructions using a Medimachine (BD Biosciences, San Jose, CA, USA). The cell suspension was then passed through a 70µm cell strainer and centrifuged. The uppermost layer was retrieved, layered on Ficoll-Hypaque separation solution, and LILs isolated by density gradient centrifugation. Viability was determined by trypan blue exclusion. For determining the differences in the frequency of CCR5 on CD4 and CD8 T cells in liver vs. periphery, fresh PBMCs and LILs were immediately stained with fluorochrome-conjugated antibodies listed in Supplementary Table 1.

Cell sorting and HCV peptide specific T-cell functions

CD4 and CD8 T cells were isolated by negative selection (Miltenyi biotec magnetic beads), stained for PE anti-CD4, APC anti-CD8 and BV421 CCR5 antibodies, and then sorted for CCR5+ and CCR5- cells using a BD FACS ARIA cell sorter.

Antigen specific functions of CCR5+ or CCR5- CD4 and CD8 T cells were assessed by measuring the frequency of cytokine secreting T cells by multi-parameter intracellular cytokine staining. Sorted cells were incubated for 5 days at 37 °C in 5% CO2 with either genotype specific overlapping HCV peptide pool (2μg/mL/peptide) covering the entire genome, or phorbol-12-myristate-13-acetate (PMA) (2.5 μg/mL) and Ionomycin (0.5 μg/mL) (Sigma) (positive control) or medium alone (negative controls). At day 4, cells were restimulated and further incubated for 2 h at 37 °C in 5% CO2 after which brefeldin A (Sigma) was added at a final concentration of 1 μg/mL as well as 1 μL of monensin (Golgi-Stop, BD Biosciences) at a final concentration of 1 μg/mL and incubated for an additional 10 h at 37 °C in 5% CO2. After incubation, cells were harvested and stained using the panel of surface and intracellular antibodies **(Supplementary table1)** following standard procedure as previously described [13]. Briefly, PBMCs were incubated with a saturated concentration of fluorescently labeled antibodies for 30 min and washed. To assess intracellular cytokine production, cells were then permeabilized using BD Perm/Wash™ buffer (BD Biosciences, USA), and then intracellular antibodies were added prior to fixing with 1% paraformaldehyde. Cells (minimum 30,000 events) were acquired in BD FACS Aria II flow cytometer. Data were analyzed using FlowJo version 9.7.7 (TreeStar, Inc.).

Supplementary Table S1. List of antibodies used for immunophenotyping of PBMC s and LILs.

Antibody	Flurochrome	clone	Catalog number	Company
CD3	Alexa Fluor 700	UCHT1	300324	BioLegend
CD4	BV605	OKT4	317438	BioLegend
CD8	PECy5	HIT8a	300910	BioLegend
CCR5	APC	J418F1	359122	BioLegend
CD45RO	APC-eFluor780	UCHL1	47-0457-42	Invitrogen
PD1	BV510	EH12.2H7	329932	BioLegend
CCR7	PE	G043H7	353204	BioLegend
CD57	BV421	NK-1	563896	BD Biosciences
CXCR3	PECY7	CXCR3-173	25-1831-82	Invitrogen
CD38	PE Texas Red	HIT2	MHCD3817	Invitrogen
HLADR	FITC	L243	307604	BioLegend
