# Supplementary figures and images for: CCR5+ T-Cells Homed to the Liver Exhibit Inflammatory and Profibrogenic Signatures in Chronic HIV/HCV-Coinfected Patients
Source: Viruses. 2021 Oct 14;13(10):2074. doi: 10.3390/v13102074 (PMC8539814; doi:10.3390/v13102074)

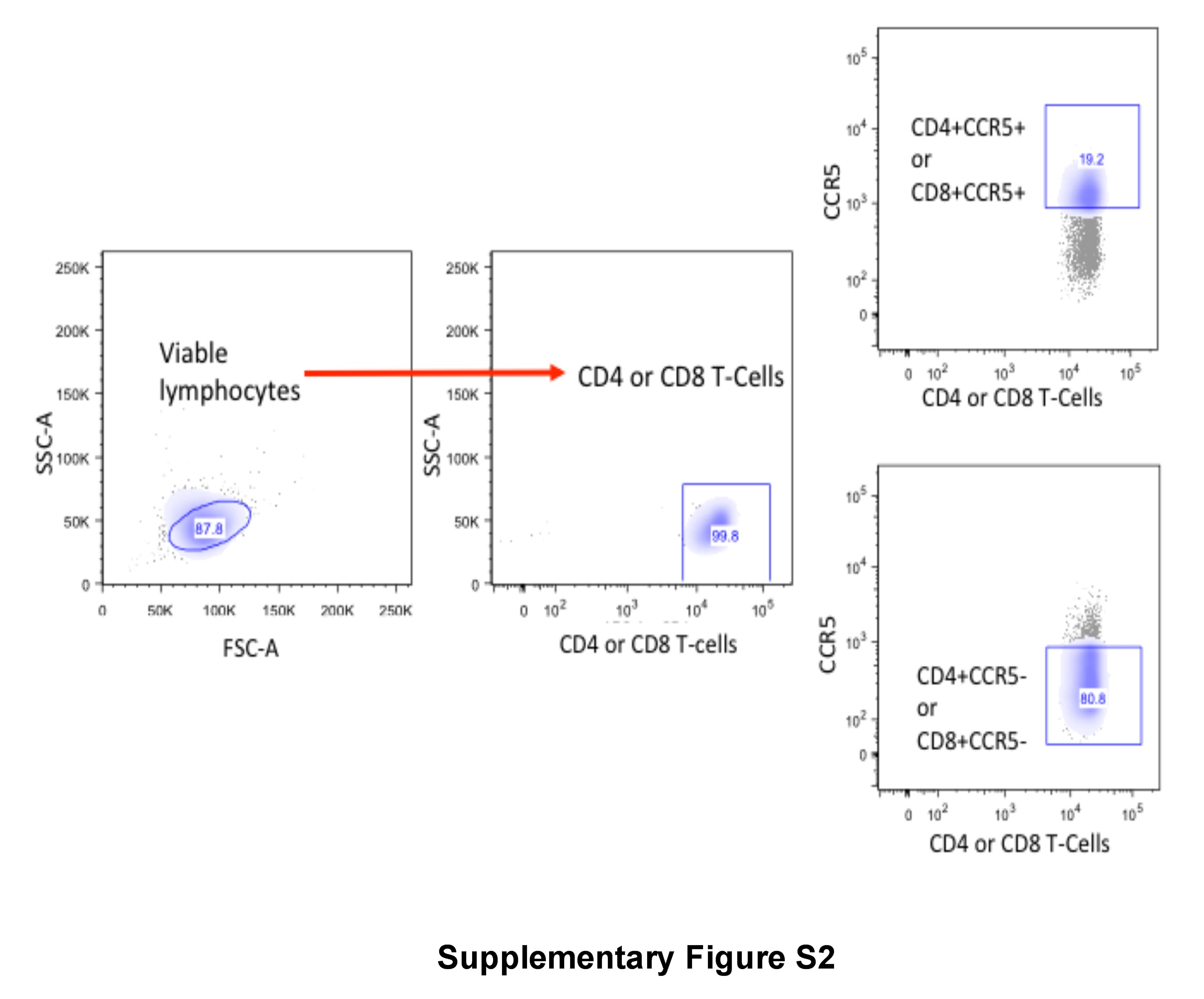

Supplement: Supplementary file 1 [file viruses-13-02074-s001.zip › Shrivastava et al. Supplementary figure s2 Viruses.jpg]

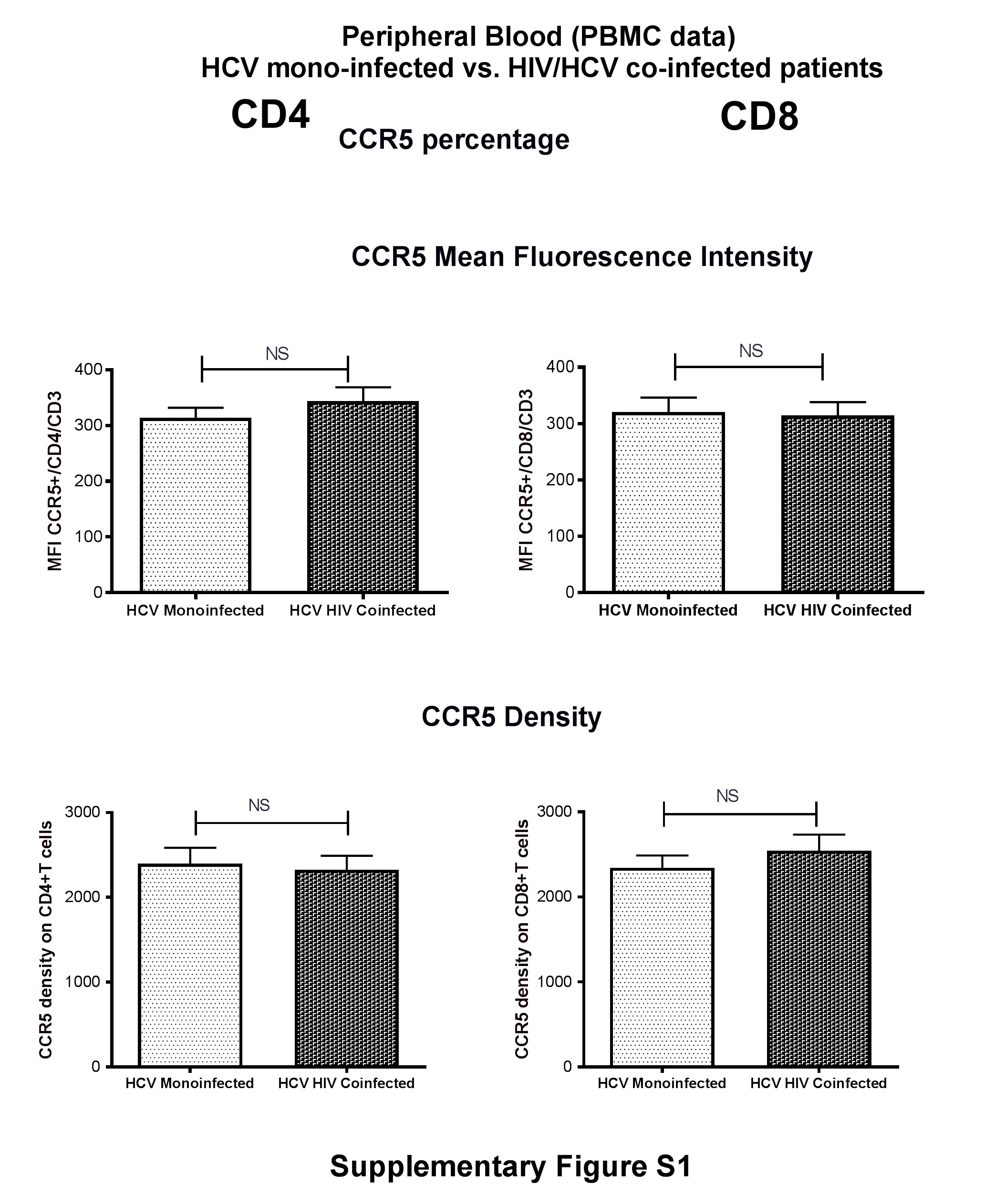

Supplement: Supplementary file 1 [file viruses-13-02074-s001.zip › Shrivastava Supplementary figure s1 Viruses.jpg]
